# A minimally invasive management for abdominal compartment syndrome in severe acute pancreatitis

**DOI:** 10.12669/pjms.291.2721

**Published:** 2013

**Authors:** Han-kui Hu, Xiao-jiong Du, Ang Li, Neng-wen Ke, Wei-ming Hu

**Affiliations:** 1 Han-kui Hu, MD, Department of Hepato-Biliary-Pancreatic Surgery, West China Hospital, 37 GuoXue Rd, Chengdu 610041, Sichuan Province, China.; 2 Xiao-jiong Du, MD, Department of Hepato-Biliary-Pancreatic Surgery, West China Hospital, 37 GuoXue Rd, Chengdu 610041, Sichuan Province, China.; 3 Ang Li, MD, Department of Hepato-Biliary-Pancreatic Surgery, West China Hospital, 37 GuoXue Rd, Chengdu 610041, Sichuan Province, China.; 4 Neng-wen Ke, MD, Department of Hepato-Biliary-Pancreatic Surgery, West China Hospital, 37 GuoXue Rd, Chengdu 610041, Sichuan Province, China.; 5 Wei-ming Hu, MD, Department of Hepato-Biliary-Pancreatic Surgery, West China Hospital, 37 GuoXue Rd, Chengdu 610041, Sichuan Province, China.

**Keywords:** Severe acute pancreatitis, Abdominal compartment syndrome, Multiple organ dysfunction syndrome, Decompression

## Abstract

Three patients with severe acute pancreatitis (SAP) developed into overt abdominal compartment syndrome (ACS) and confirmed or suspected infection of necrotic tissue. We successfully treated these patients by minimally invasive decompression with the assist of laparoscope after the failures of intensive care treatments. This technique we report here may be another safe and effective management for ACS in SAP.

## Introduction

 Abdominal compartment syndrome (ACS) in severe acute pancreatitis (SAP) has been recognized closely related to considerable mortality rate.^1^^-^^3^ When conservative measures fail to decrease intra-abdominal pressure (IAP) and control worsening organ dysfunction, surgical intervention is needed instantly.^4^ Several surgical approaches reported recently have shown their effectiveness, however, each treatment has imperfections, so safer and more effective procedures are crying needed.

## Case Reports and Surgical Technique

 Three patients with confirmed SAP, included a 27-year-old man with cholelithiasis (Case 1), a 60-year-old woman with hyperlipidemia (Case 2), a 45-year-old woman with hyperlipidemia (Case 3), were admitted to the surgical intensive care unit (SICU) because of severe abdominal distension, ongoing renal failure and respiratory failure at 29, 8, 6 days after onset, respectively. However, percutaneous drainage, pressure-control ventilation, hemodiafiltration, and other conservative treatments failed to decrease IAP and control worsening organ dysfunction.

**Fig.1:**
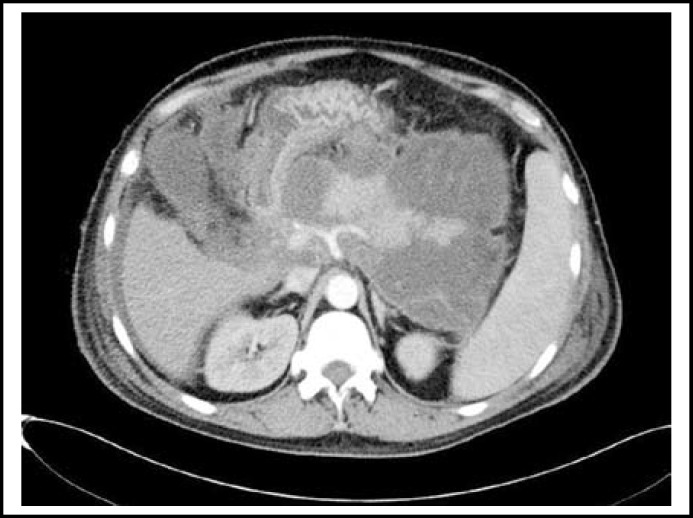
CT confirmed the collections of ﬂuid, and necrotic tissue in the peripancreatic area of Case 1

 Two-five days later of SICU admission, Contrast-enhanced Computed tomography (CT) confirmed the collections of ﬂuid, and necrotic tissue in the peripancreatic area of Case 1 (Fig.1) and 3, a mass of blood and necrotic tissue in the peripancreatic area of Case 2; drainage culture confirmed the existence of gram negative bacteria in Case one, sepsis was found in Case two and three; immature neutrophils showed a growing trend, body temperature rose above 38 degrees C of the three patients; IAP (measured via Foley catheter) ranged from 22-26mmHg; Urine output less than 160ml; tidal volume ranged from 290-340 ml; PaO2/FiO2 ranged from 158-204; Peak airway pressure (PAP) maintained more than 38 cmH2O; APACHE II score more than 16. The patients were brought to the operating room to undergo decompression for ACS. Comparing to the preoperative day, the IAP decreased markedly, the urine output increased to more than 3000ml on the 7th postoperative day, tidal volume, Peak airway pressure (PAP) and PaO2/FiO2 was improved satisfactorily (Table-I). Via 15, 11 and 10 days standard intensive care treatment, respectively, respiratory and renal function of the patients recovered completely and were transferred to general ward. 

**Table-I:** Clinical data of the 3 patients

			*IAP*	*Urine output*	*VT*	*PaO2/FiO2*	*PAP*	*APACHE II*
Case 1	pre.		26	70	320	158	49	21
	post.	1d	15	510	470	245	29	15
		3d	13	1500	490	300	26	10
		7d	10	3000	510	348	19	6
Case 2	pre.		25	110	290	195	43	18
	post.	1d	17	450	450	269	27	13
		3d	15	2000	500	320	21	9
		7d	9	3400	500	345	17	5
Case 3	pre.		22	160	340	204	38	16
	pos.	1d	14	540	440	317	21	11
		3d	11	1800	510	360	18	7
		7d	9	3100	520	382	16	5

Pre.: Preoperation, Post.: Postoperation, IAP: Intra-abdominal pressure (mmHg),

VT: Tidal Volume (ml), PAP: Peak airway pressure (cmH2O).

 All the patients had postoperative lavage, drainage pipes were pulled out gradually when efﬂuent less than 5ml and no evidence of fluid and infection of necrotic tissues in peripancreatic area. For the formation of abscess in fossa iliaca, Case one experienced repeated debridement via the wound at 8 postoperative days under local anesthesia. After 71, 54 and 31 days of postoperative hospitalization, respectively, the three patients were discharged and recovered completely without any complication within the next two months.

**Fig.2:**
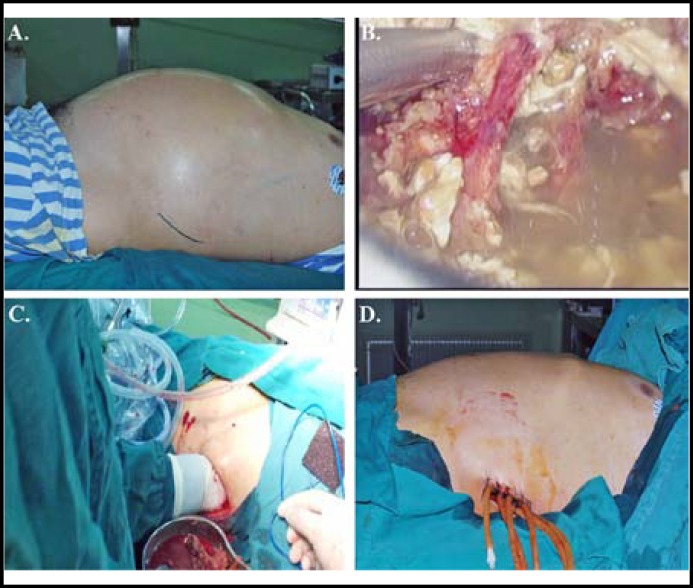
A: Location of the incision.

 The operation was performed into the following 5 steps : 1) Under general anesthesia, with lateral position about 30 degrees, a 6 cm long oblique incision from the root of the 12th rib and lateral of the erector spinae was made in the left or both sides, the subcutaneous tissue was incised (Fig.2A). 2) A 12-mm and a 6-mm trocars were inserted into retroperitoneum, maintained the gas (CO2) pressure at 10 mmHg; inspected the retroperitoneum, aspirated necrotic tissue, blood clot and efﬂuent under laparoscopic control (Fig.2B). 3) Opened the muscle layers, debrided necrotic tissue in retroperitoneum and omental sac gently with single hand (Fig.2C). 4) Lavaged retroperitoneum and omental sac and controlled the bleeding with laparoscopy. 5) Four-six drainage tubes were placed in peripancreatic area and fossa iliaca via the wound which was laid open (Fig.2D).

B: Inspected the retroperitoneum, aspirated necrotic tissue, blood clot and efﬂuent under laparoscopic control.

C: Debrided necrotic tissue in retroperitoneum and omental sac gently with single hand.

D: Drainage tubes were placed in peripancreatic area and fossa iliaca via the open wound.

## Discussion

 ACS in SAP has been recognized closely related to considerable mortality rate. It is thought to be one speciﬁc group of candidates for surgery when conservatively approach is non-effective for ACS in SAP^4^; if there are evidences of infected necrotic tissue, patients may undergo debridement.^5^^,^^6^ There have not been standard surgical management, nowadays, several surgical approaches reported recently have shown their effectiveness, however, each treatment has imperfections. Traditional laparotomy is associated with high rates of uncontrollable hemorrhage, intestinal fistula and infection in enterocoelia and considerable resource utilization^7^; Direct Retroperitoneal Open Drainage via a Long Posterior Oblique Incision reported by Zenichi Morise in Japan which could decrease IAP, but it was hard to control the bleeding from a blind area and would be difficult for postsurgical management^8^; Transverse laparostomy always leads to the loss of abdominal and back extensor muscle functions^9^; subcutaneous linea alba fasciotomy often results in ventral hernia requiring abdominal wall reconstruction.^10^

 The approach we report here may cover the HYPERLINK “app:ds:imperfection”imperfections above：1. With the assistance of laparoscope, we can control hemorrhage from a blind area. 2. This is a minimally invasive approach, allowing repeated debridement via the wound after surgery, without loss of abdominal and back extensor muscle functions. 3. Compared with laparotomy, it reduces the risk of intestinal ﬁstulas, intraabdominal infection and the difficulty of postsurgical nursing care. 4. The whole retroperitoneal area can be debrided by single hand within short time. 5. Drainage tubes placed in retroperitoneum and the open wound will be sufficient for drainage, avoiding recurrent ACS. 6. Postoperative lavage can remove necrotic tissue persistently and prevent infection. While, this method may not be applicable for all kinds of ACS in SAP, whether this procedure increase the risk of infection in patients with aseptic necrotic tissues is unknown, sufficient space between colon and kidney allowing to approach the whole retroperitoneal area is a necessary requirement.

 In summary, we successfully treated the three patients with this approach; it may be another safe and effective management for ACS in SAP. Prospective studies are needed to compare this technique with others.
